# Current Evidence on the Relationship Between Perioperative Hypothermia and Surgical Site Infection: A Scoping Review

**DOI:** 10.3390/jcm15124501

**Published:** 2026-06-10

**Authors:** Angie Paola Ortiz-Tello, Sebastian Ospina-Gomez, Nicole Bonilla, Fernando Ríos-Barbosa, Eduardo Tuta-Quintero

**Affiliations:** 1 Department of Anesthesiology, School of Medicine, Universidad de La Sabana, Campus del Puente del Común, Km. 7, Autopista Norte de Bogotá, Chía 250001, Colombia; angieorte@unisabana.edu.co (A.P.O.-T.); sebastianosgo@unisabana.edu.co (S.O.-G.); nicolebosi@unisabana.edu.co (N.B.); 2 Department of Epidemiology, School of Medicine, Universidad de La Sabana, Campus del Puente del Común, Km. 7, Autopista Norte de Bogotá, Chía 250001, Colombia; eduardotuqu@unisabana.edu.co

**Keywords:** hypothermia, infection, surgery, systematic review

## Abstract

**Background**: Surgical site infection (SSI) is a common complication that increases morbidity, prolongs hospital stays, and raises healthcare costs. Perioperative hypothermia may contribute to its development by altering tissue perfusion, oxygenation, and the immune response. **Objective**: To evaluate the association between perioperative hypothermia and the risk of SSI in adult patients. **Methods**: A scoping review was conducted following the methodological framework of Arksey and O’Malley, expanded by Levac, the recommendations of the Joanna Briggs Institute, and the PRISMA-ScR guidelines. A systematic search was performed in PubMed and Scopus through 30 April 2026. We included observational studies and clinical trials in adults undergoing surgery that evaluated the association between perioperative or intraoperative hypothermia and the occurrence of SSI or other postoperative infectious complications. **Results**: A total of 28 studies were included. Retrospective observational studies were the most common design, comprising 19/28 studies (67.9%), followed by 6/28 prospective cohorts (21.4%) and 4/28 randomized clinical trials (14.3%). The studies were conducted across 12 countries, with the United States contributing the largest proportion (14/28, 50%), followed by China, Turkey, and Japan with two studies each (7.1% per country). Regarding the main findings, 14 studies (50%) reported a positive association between perioperative hypothermia and an increased risk of SSI or other postoperative infections, whereas 11 studies (39.3%) found no statistically significant association. Most studies (15/28, 53.6%) used a fixed temperature threshold of <36 °C, while a smaller proportion applied lower cutoffs such as <35–35.5 °C (3/28, 10.7%); in the remaining studies (10/28, 35.7%), the threshold was not clearly specified. Temperature measurement methods were frequently underreported (21/28, 75.0%). Among studies that did report them, approaches included repeated measurements (3/28, 10.7%), continuous monitoring (2/28, 7.1%), mean intraoperative temperature (1/28, 3.6%), nadir temperature (1/28, 3.6%), and single-point measurements (1/28, 3.6%). **Conclusions**: Perioperative hypothermia may be associated with an increased risk of SSI; however, the available evidence is inconsistent across surgical settings. Rather than indicating a clear independent effect, the findings suggest that hypothermia could play a context-dependent role within a broader set of perioperative factors influencing infection risk.

## 1. Introduction

Surgical site infection (SSI) is a common postoperative complication in patients undergoing surgery, which can lead to increased morbidity, prolonged hospital stays, the need for reoperation, and higher healthcare costs [1,2]. SSIs occur particularly in major procedures involving multiple perioperative factors, which may be modifiable [2,3]. Among these factors, the maintenance of normothermia stands out as a protective factor, thereby highlighting some controversy regarding the role of perioperative hypothermia as an independent risk factor, given the alteration of the microenvironment and the resulting inflammatory cascades [4,5].

Exposure to temperature-controlled surgical environments, general or regional anesthesia, the administration of cold intravenous fluids, and the prolonged duration of procedures promote heat loss and the development of unnoticed perioperative hypothermia, typically defined as a core temperature below 36 °C [5,6]. Hypothermia can induce peripheral vasoconstriction, reduce tissue perfusion, decrease tissue oxygen tension, and alter cellular immune function—conditions that may compromise the host’s ability to prevent bacterial colonization of the surgical wound [6,7].

The inflammatory cascade triggered by hypothermia can suppress leukocyte function and exacerbate a dysregulated inflammatory response [5,6,7]. Current evidence has shown that patients who develop intraoperative hypothermia present a significantly higher incidence of surgical site infections compared with those maintained at normothermia [5,6,7,8]. However, other observational studies with significant methodological differences have shown heterogeneous results, particularly in abdominal, orthopedic, colorectal, and oncological surgery [8,9]. Furthermore, there is no control for confounding variables such as the degree of hypothermia achieved, the duration of thermal exposure, temperature measurement methods, and surgical duration [10]. Therefore, it is pertinent to review the existing scientific evidence regarding the association between perioperative and intraoperative hypothermia and the incidence of surgical site infection or other postoperative infectious complications in patients undergoing surgical procedures.

## 2. Methods

A scoping review was conducted following the methodological framework proposed by Arksey and O’Malley [11] and expanded by Levac [12], as well as the Joanna Briggs Institute’s [13] methodological guidelines for scoping reviews. The report was prepared in accordance with the PRISMA extension for exploratory reviews (PRISMA-ScR) (Supplementary File) [14]. The review was conducted using a systematic approach in five consecutive stages: (1) formulation of the research question, (2) identification of relevant studies, (3) selection of studies according to predefined criteria, (4) standardized data extraction, and (5) synthesis and presentation of the findings.

### 2.1. Research Question

The central question guiding this review was: What is the available evidence regarding the association between perioperative and intraoperative hypothermia and the incidence of surgical site infection or other postoperative infectious complications in patients undergoing surgical procedures?

### 2.2. Elegibility Criteria

Included studies had to involve adult, non-pregnant patients undergoing surgical procedures, assessing the presence of perioperative or intraoperative hypothermia (unintentional or related to thermal management strategies), and analyze its association with surgical site infection or other postoperative infectious complications. Observational studies and randomized clinical trials published in English or Spanish were considered. Studies conducted across different surgical specialties and clinical settings were included, provided they reported infectious outcomes related to perioperative body temperature. Studies focused exclusively on warming devices without a direct assessment of the effect of hypothermia on infection, articles without access to the full text, protocols, editorials, commentaries, and studies that did not clearly identify the relationship between thermal exposure and infectious outcomes were excluded.

The operational definition of hypothermia used in this review was classified into three main approaches:

(1) fixed temperature thresholds (generally <36 °C, but with a range of <35 to <35.5 °C),
(2) time-dependent definitions based on the duration of exposure below a given threshold, and
(3) composite or continuous measures, such as cumulative heat load or area under the temperature curve.

### 2.3. Information Sources and Search Strategy

Systematic research was conducted in two electronic databases: PubMed, Embase and Scopus. The search strategy was developed using MeSH terms linked by Boolean operators (Supplementary File). The search included articles published up to 30 April 2026.

### 2.4. Study Selection Process

Search results were exported and organized in RIS formats, and duplicates were removed manually and using a bibliographic manager. Subsequently, the records were imported into Rayyan [15], a platform where two independent reviewers assessed titles and abstracts according to previously standardized eligibility criteria. Studies classified as “doubtful” or those with discrepancies underwent a second review; if disagreement persisted, a third reviewer determined their inclusion or exclusion. The selected studies proceeded to full-text evaluation following the same procedure. The entire process was documented using a PRISMA-ScR flowchart [14].

### 2.5. Data Extraction

Information was extracted by two independent reviewers using a structured template that included the following variables: author, authors’ country, study design, study objective, main results, and reported limitations. The findings were synthesized through a descriptive summary of the characteristics of the included studies and a narrative synthesis organized by study design, in accordance with the categories proposed by Grudniewicz and colleagues [16]. In addition, heterogeneity in the definition and measurement of perioperative hypothermia was assessed by extracting and categorizing key methodological variables from each included study. Specifically, temperature thresholds, methods of temperature measurement, and exposure metrics were recorded. We documented whether SSI was evaluated as a primary or secondary outcome.

## 3. Results

**Figure 1:**
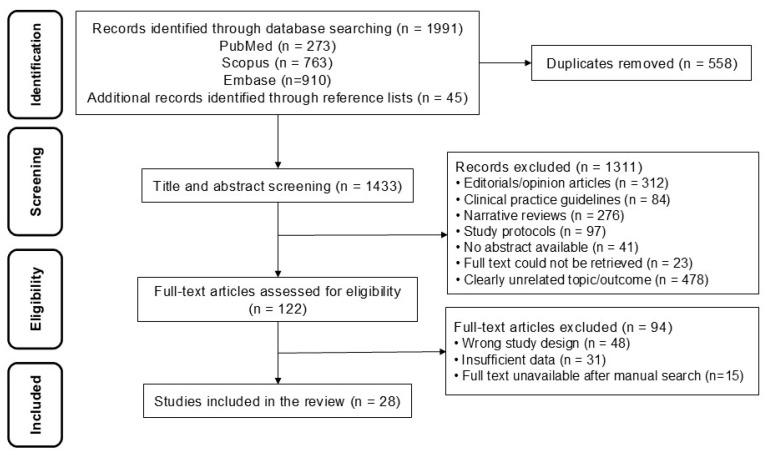
PRISMA-ScR flow diagram.

A total of 28 studies were included that evaluated the association between perioperative hypothermia and the risk of postoperative infection (Figure 1). In terms of methodological design, retrospective observational studies were the most common, with 19/28 studies (67.9%) classified as retrospective cohorts, followed by 6/28 prospective cohorts (21.4%) and 4/28 randomized clinical trials (14.3%). The studies were conducted across 12/28 countries, with the United States being the most represented, contributing 14/28 studies (50%), followed by China, Turkey, and Japan with 2/28 studies each (7.1% each). The remaining studies were conducted in Serbia, Brazil, Canada, Mexico, Republic of Korea, Thailand, Israel, and Pakistan, with one study each (Table 1).

Regarding the main findings, 14/28 studies (50%) reported a positive association between hypothermia and an increased risk of surgical site infection or other postoperative infections [17,25,28,31,33,37]. In contrast, 11/28 studies (39.3%) found no statistically significant association [20,23,24,42,43]. Additionally, 3 studies (10.7%) reported indirect, inconclusive, or context-dependent results, primarily related to immunological changes, warming strategies, or infection outcomes not defined as primary endpoints, such as in randomized trials where infection was reported only as a secondary or safety outcome [19,41,44]. The definition of hypothermia varied across studies, with most using a threshold of <36 °C, while others applied lower cutoffs or incorporated dynamic measures such as duration of exposure and cumulative thermal burden [18,21,25,28,31,33].

The operational definition and measurement of hypothermia showed substantial variability across studies. Three main approaches were identified: (1) fixed temperature thresholds, most commonly <36 °C, although several studies used lower cutoffs such as <35–35.5 °C [18,21,25,28,33]; (2) time-dependent definitions based on the duration of exposure below a given threshold (e.g., >75, >90, or >195 min) [21,25,29,31]; and (3) composite or continuous measures, including cumulative thermal burden or area under the temperature curve [21,31]. Additionally, temperature monitoring methods were inconsistently reported and likely varied across studies, including differences in measurement site and frequency of recording [17,20,22].

SSI was defined as the primary outcome of interest, while other postoperative infectious complications (e.g., pulmonary, systemic, or unspecified infections) were considered secondary outcomes [17,25,28,37]. Outcomes relevant to the research question were assessed accordingly across the included studies. Additional outcomes included deep or systemic infections, postoperative pulmonary infection, general infectious complications, and changes in inflammatory immune markers secondary to the humoral effects of perioperative hypothermia [19,21,26,29,39,41]. Regarding methodological limitations, the following were repeatedly identified: a predominance of single-center studies, small sample sizes, retrospective observational designs, a lack of standardization in body temperature measurement, and insufficient control of perioperative confounding variables [17,20,22,23,34,36]. These factors may affect comparability, homogeneity across studies, and the generalizability of results, highlighting the need for greater consistency in the current evidence to support the development of standardized management guidelines.

The duration and cumulative exposure to hypothermia were evaluated in multiple studies [21,25,29,31,33], most of which reported a significant association with infectious outcomes, including surgical site infections and other postoperative infections. Prolonged exposure to hypothermia was consistently associated with increased risk [21,29,31]. Several studies also suggested stronger associations at lower temperature thresholds (≤35–35.5 °C), supporting a potential dose–response relationship [18,31,33].

A more consistent association was observed in abdominal, oncologic, and burn-related procedures [23,25,29,31,33], whereas studies in orthopedic surgery [22,42,43] generally did not demonstrate a significant relationship. Procedure-related factors such as prolonged surgical duration and cumulative hypothermic exposure [21,29,31] appeared to increase risk, while patient-related factors, particularly diabetes and poor glycemic control [34], showed a potential additive effect. Additionally, the role of temperature management strategies was heterogeneous; while some studies suggested that active warming may mitigate risk, others focused primarily on thermal exposure rather than specific interventions, highlighting variability in clinical practice [20,35].

Zhou YD et al. [20] a retrospective cohort study in which they analyzed the possible correlation between unnoticed perioperative hypothermia and surgical site infection following liver resection. The results showed that there is no significant association between mild perioperative hypothermia and surgical site infection. This could be explained by adequate management of perioperative temperature, particularly through active warming measures, which limits the impact of hypothermia on the development of SSI.

Pang QY et al. [21] conducted a retrospective cohort study in which they evaluated the relationship between hypothermia (<36 °C) and intraoperative hyperthermia (>37.3 °C), considering absolute temperature values, duration of exposure, and area under the curve, with the occurrence of postoperative pulmonary infection (PPI) and surgical site infection (SSI) in major non-cardiac surgery. They found that the duration and area under the curve of intraoperative hypothermia can predict postoperative pulmonary infection and surgical site infection; however, absolute temperature values were not associated with these outcomes. Therefore, they suggest that future studies focus on improving intraoperative body temperature monitoring to prevent hypothermia and hyperthermia.

Ribeiro JC et al. [25], in a prospective cohort study of patients undergoing abdominal surgery, reported a surgical site infection rate of 20.25% and found a higher likelihood of developing a surgical site infection (RR = 1.89) in patients with temperatures < 36 °C for more than 75 min or multiple episodes of hypothermia, establishing hypothermia as an independent risk factor for surgical site infection.

Andersen et al. [28] in a retrospective study of patients undergoing breast reconstruction with implants, demonstrated that intraoperative hypothermia (<35.5 °C) is associated with a higher rate of surgical site infection compared to normothermic patients; as well as an increase in healing complications, with a higher probability of developing surgical site infection and delayed healing. Furthermore, a longer duration of hypothermia was significantly associated with the onset of infection. They therefore suggest that maintaining strict normothermia during procedures may improve patient outcomes by reducing the risk of postoperative infection and delayed wound healing.

### Heterogeneity in the Definition and Measurement of Perioperative Hypothermia

Most studies (15/28, 53.6%) defined hypothermia using a fixed temperature threshold of <36 °C, while a smaller proportion used lower cutoffs such as <35–35.5 °C (3/28, 10.7%); in the remaining studies (10/28, 35.7%), the threshold was not clearly specified (Table 2). Measurement methods were frequently not reported (21/28, 75.0%); among those that did, approaches included repeated measurements (3/28, 10.7%), continuous monitoring (2/28, 7.1%), mean intraoperative temperature (1/28, 3.6%), nadir temperature (1/28, 3.6%), and single measurements (1/28, 3.6%). Regarding exposure metrics, dichotomous definitions (presence vs. absence of hypothermia) were the most common approach (16/28, 57.1%). More complex metrics were used less frequently, including duration-based definitions (3/28, 10.7%), percentage of operative time (1/28, 3.6%), continuous measures such as temperature change per degree (n = 3, 10.7%), and composite metrics such as area under the curve (AUC) (1/28, 3.6%). SSI was assessed as the primary outcome in most studies (22/28, 78.6%), while a minority reported SSI as a secondary outcome or evaluated indirect outcomes such as immune response (6/28, 21.4%).

## 4. Discussion

This scoping review maps the existing literature on the relationship between perioperative hypothermia and SSI, highlighting the breadth, variability, and limitations of the available evidence. The included studies were heterogeneous in both observational and randomized clinical trial designs, with approximately half reporting a potential association between hypothermia and infection, while others found no statistically significant relationship [1,2,3,5,22]. Importantly, this review identified substantial variability in how hypothermia was defined, measured, and analyzed. Some studies used fixed temperature thresholds, whereas others incorporated dynamic variables such as duration of exposure or cumulative thermal burden. In addition, SSI was inconsistently defined and reported using variable diagnostic criteria, limiting comparability across studies [21,25,29,31,33,34,35,36,37,38,39,40,41,42,43,44]. Consequently, studies categorized as evaluating “hypothermia” often assessed different types of thermal exposure, reducing the direct comparability of findings.

These findings suggest that perioperative temperature should be interpreted within a broader multifactorial clinical context that includes surgical duration, procedural complexity, hemodynamic status, antibiotic prophylaxis, and temperature management strategies [4,6]. However, given the exploratory nature of this review and the marked heterogeneity of the evidence included, causal inferences cannot be established. From a scoping review perspective, our findings identify several key gaps that should guide future research, including the need for standardized definitions of hypothermia, consistent reporting of SSI outcomes, and adequately powered randomized trials with SSI as a primary endpoint [21,25,29,31,33]. Addressing these limitations will be essential for future systematic reviews and meta-analyses aimed at quantitatively evaluating this relationship.

A central finding of this review was the lack of standardized definitions and measurement approaches for perioperative hypothermia across studies [25,28,33,34,44]. This variability directly affects the interpretation of the reported associations with SSI. Hypothermia was operationalized using different temperature thresholds, ranging from <36 °C to <35 °C, as well as dynamic measures such as duration of exposure and cumulative thermal burden. Furthermore, inconsistencies in temperature monitoring techniques and anatomical measurement sites further complicated comparisons between studies [21,25,29,31,33].

Because hypothermia was defined and measured differently across studies, the exposure of interest was not uniform throughout the literature, making direct comparisons challenging and potentially misleading [17,20,21,25,31]. As a result, studies reporting associations between hypothermia and SSI may not have been evaluating equivalent clinical phenomena [18,23,28,33]. Future investigations should prioritize harmonization of temperature thresholds, standardized monitoring techniques, and consistent reporting of exposure metrics to facilitate more robust comparisons and support high-quality systematic reviews and meta-analyses.

There is sufficient biological plausibility to support hypothermia as a potential contributor to postoperative infection. A reduction in core body temperature induces peripheral vasoconstriction, decreases tissue perfusion, and lowers local oxygen tension, thereby impairing neutrophil bactericidal activity and innate immune function [41]. In addition, hypothermia alters enzymatic activity within the inflammatory cascade and modifies cytokine release, potentially compromising tissue healing and facilitating bacterial colonization [19,39,41]. These pathophysiological mechanisms are consistent with classic findings such as those reported by Kurz et al., who demonstrated a lower incidence of surgical infection among patients maintained under intraoperative normothermia [38].

The clinical impact of hypothermia appears to vary according to the type of surgery, procedural duration, nadir temperature, and the effectiveness of intraoperative temperature correction. Zhou et al., in patients undergoing liver resection, found no significant association between inadvertent perioperative hypothermia and SSI, possibly due to the systematic use of active warming strategies [20]. In contrast, Ribeiro et al. reported that temperatures below 36 °C maintained for more than 75 min significantly increased infection risk, suggesting a potential thermal dose-response relationship [25]. Similarly, Pang et al. found that the duration of hypothermia and the area under the thermal curve were stronger predictors of postoperative pulmonary infection and SSI than absolute temperature values alone [21]. These findings are consistent with recent studies proposing a shift beyond the conventional dichotomous classification of hypothermia (<36 °C vs. ≥36 °C) toward continuous thermal exposure metrics [1,4].

The heterogeneity in the operational definition of hypothermia remains one of the principal sources of inconsistency across studies. Andersen et al., for example, identified a significant association between temperatures below 35.5 °C and a higher incidence of infection and delayed wound healing in implant-based breast reconstruction [28]. This finding suggests that lower temperature thresholds may carry greater clinical relevance, particularly in procedures involving microvascular compromise or complex reconstruction. Similar observations have been reported in abdominal oncologic surgery, burn care, and prolonged colorectal procedures [26,29,31].

Physiological variability among surgical procedures also limits the universal generalization of these findings. The physiological characteristics of liver resection, colectomy, orthopedic surgery, and breast reconstruction differ considerably in terms of tissue exposure, blood loss, operative duration, anesthetic management, and baseline infection risk [7,23,30,34]. This variability may explain why some orthopedic studies did not identify a clear association between hypothermia and infection [22,42,43], whereas the association appeared more evident in abdominal surgery and prolonged procedures [24,25,33].

Finally, the predominance of retrospective observational studies limits the ability to establish causality. Approximately two-thirds of the included studies were retrospective cohorts, making them susceptible to selection bias, reporting bias, and incomplete adjustment for perioperative confounding variables [3,34,36]. In this context, some authors have suggested that hypothermia may function as an indirect marker of surgical complexity or overall physiological vulnerability rather than as an isolated causal mechanism [5,34]. This interpretation is supported by studies such as that of Pang et al., in which the association with postoperative pulmonary infection suggested a broader systemic process rather than a purely localized infectious phenomenon [21].

### Limitations

One of the main limitations of this review was the absence of a formal assessment of risk of bias and the lack of a meta-analysis, which prevents a quantitative assessment of the methodological quality of the included studies and the estimation of an overall effect size between perioperative hypothermia and surgical site infection. Since this was an exploratory review using a scoping review methodology, the primary objective was to map the available evidence rather than establish definitive causal relationships [11,12,13,14]. Finally, the search was restricted to PubMed and Scopus and included only studies in English and Spanish; therefore, it is possible that relevant evidence in other languages or databases was not identified.

The decision to conduct a scoping review was driven not only by heterogeneity, but also by the conceptual variability in how perioperative hypothermia is understood and operationalized across studies. Several investigations incorporate dynamic measures such as duration of exposure or thermal burden, while others assess indirect immunological or systemic effects rather than strictly defined SSI outcomes [17,18,19,20,21,22,23,24,25,26,27,28,29,30,31,32,33,34,35,36,37,38,39,40,41,42,43]. This variability reflects an evolving field in which key constructs and measurement approaches are not yet standardized. In addition, although randomized clinical trials were included, surgical site infection was frequently reported as a secondary or exploratory endpoint rather than a primary outcome. As a result, many of these trials were not specifically powered to detect differences in SSI, which may limit the strength and comparability of their findings. Conversely, retrospective cohort studies often reported SSI as a primary or explicitly analyzed outcome but are inherently more prone to bias and confounding. The inclusion of both study de-signs, while necessary to comprehensively map the available evidence, introduces an imbalance in the level of evidence and may affect the overall interpretation of the association between perioperative hypothermia and infection risk.

The analyzed evidence was dominated by retrospective observational studies, which increases susceptibility to methodological biases, particularly selection bias, reporting bias, and incomplete control of confounding factors such as surgical duration, bleeding, prophylactic antibiotics, anesthetic technique, and active warming strategies [3,23,34].

## 5. Conclusions

Perioperative hypothermia may be associated with an increased risk of SSI, although the available evidence remains heterogeneous and inconclusive. Current findings suggest that hypothermia may function as a modulating factor within a multifactorial perioperative context rather than as an independent determinant. While maintaining normothermia continues to be recommended in clinical practice, further well-designed and standardized studies are needed to better characterize its relationship with infectious outcomes.

## Figures and Tables

**Table 1:** General characteristics of the included articles.

Author	Authors’ Country	Study Design	Objective	Results	Limitations
Yilmaz Eker, et al. [17]	Turkey	Prospective cohort	To determine whether unnoticed perioperative hypothermia affects the risk of surgical site infection in patients undergoing bariatric surgery.	The presence of perioperative hypothermia following bariatric surgery was associated with an increased incidence of surgical site infections (*p* = 0.001).	Lack of randomization. Variation in how temperature was measured and managed.
Walters, MJ, et al. [18]	United States	Retrospective cohort	To assess whether there is a relationship between the average intraoperative core temperature and the risk of serious infections (local or systemic) in adults undergoing colorectal surgery under general anesthesia	When the average temperature was ≤35.4 °C, each 0.5 °C decrease was associated with a higher probability of severe infection (OR = 1.38; *p* = 0.045).	Single-center study. Small sample size.
Zeba, S, et al. [19]	Serbia	Randomized clinical trial	To assess how intraoperative hypothermia affects the cytokine profile (inflammatory markers) in surgical patients.	Intraoperative warming attenuated the increased and sustained proinflammatory response, which is potentially harmful, present in the unwarmed controls (*p* < 0.01).	Single-center study. Small sample size.
Zhou YD, et al. [20]	China	Retrospective cohort	To assess whether unnoticed perioperative hypothermia is associated with an increased incidence of surgical site infections following liver resection.	No significant association was found between hypothermia and SSI following liver resection: high exposure to hypothermia (OR = 1.25; 95% CI: 0.84–1.87; *p* = 0.266), moderate exposure (OR = 1.00; 95% CI: 0.65–1.53; *p* = 0.999), and low exposure (OR = 1.11; 95% CI: 0.73–1.65; *p* = 0.628).	Non-standardized temperature measurement and thermal management. Clinical variation in real-world practice.
Pang QY, et al. [21]	China	Retrospective cohort	Association between intraoperative hypothermia and hyperthermia with postoperative pulmonary infection and surgical site infection in major non-cardiac surgery.	Intraoperative hypothermia and hyperthermia were found to be associated with an increased risk of postoperative pulmonary infection related to the duration of exposure (hypothermia > 90 min: aOR = 1.425; 95% CI: 1.131–1.796; hyperthermia > 75 min: aOR = 1.395; 95% CI: 1.208–1.612), AUC for hypothermia (aOR 1.390) and hyperthermia (aOR 2.045), and with surgical site infection also associated with duration (hypothermia > 195 min: aOR = 2.900; 95% CI: 1.703–4.937; hyperthermia > 75 min: aOR = 1.395; 95% CI: 1.208–1.612), AUC for hypothermia (aOR 2.665) and hyperthermia (aOR 2.619) in major non-cardiac surgery.	Variability in temperature measurement. Limited generalizability if it is a single-center study.
Abugri BO, et al. [22]	Japan	Retrospective cohort	Association between Surgical Site Infection and Intraoperative Hypothermia in Total Hip and Knee Arthroplasties.	Intraoperative hypothermia occurred in 18.8% of patients and was not associated with SSI in adults undergoing total hip and knee arthroplasty. In contrast, temperatures > 36 °C were associated with an increased risk of SSI (OR = 3.6; 95% CI: 1.367–9.475; *p* = 0.009).	Single-center study.
Baucom RB, et al. [23]	United States	Retrospective cohort	To determine whether intraoperative hypothermia in patients undergoing segmental colectomy is associated with postoperative surgical site infection	Patients undergoing segmental colectomy who experienced a period of intraoperative hypothermia were no more likely to develop a surgical site infection than those who were normothermic (OR = 1.17; 95% CI, 0.76–1.81; *p* = 0.48).	Single-center study. Specific colorectal surgery. Does not evaluate deeper hypothermia (<35 °C).
Siddiqiui T, et al. [24]	United States	Prospective cohort	To determine the association between hypothermia and surgical site infection in elective abdominal surgery.	No statistically significant association was found between hypothermia and surgical site infection, with a similar SSI rate in patients with and without hypothermia (10% vs. 10.8%; *p* = 0.867).	Observational study Small sample size
Ribeiro JC, et al. [25]	Brasil	Prospective cohort	To determine the independent association between perioperative hypothermia and the incidence of surgical site infection in patients undergoing abdominal surgery.	Perioperative hypothermia was an independent risk factor for surgical site infection (RR = 1.89)	Single-center study
Tsuchida T, et al. [26]	United States	Retrospective cohort	To determine whether unintentional perioperative hypothermia is associated with an increased risk of postoperative infection.	Severe hypothermia and delayed hypothermia were associated with a higher incidence of surgical site infection and organ/space infection; however, they were not identified as independent risk factors for SSI in the multivariate analysis (severe hypothermia: OR = 1.24; 95% CI: 0.56–2.77; late-onset hypothermia: OR = 0.71; 95% CI: 0.46–1.01).	Small sample size
Frisch NB, et al. [27]	United States	Retrospective cohort	To evaluate the effect of intraoperative hypothermia on complications and clinical outcomes in patients with hip fractures undergoing surgical treatment.	Intraoperative hypothermia was associated with an increased rate of deep surgical site infection (OR = 3.30; 95% CI: 1.19–9.14; *p* = 0.022); in addition, a lower body mass index (*p* = 0.004) and older age (*p* = 0.005) were identified as risk factors for hypothermia.	Small sample size
Andersen ES, et al. [28]	United States	Retrospective cohort	To analyze the association between intraoperative hypothermia, as a modifiable risk factor, and the occurrence of postoperative surgical site infection in patients undergoing immediate breast reconstruction with implants following mastectomy.	Intraoperative hypothermia is a significant risk factor for postoperative infection in breast reconstruction with implants following mastectomy (OR = 2.567; 95% CI: 1.367–4.818; *p* < 0.05), and delayed wound healing (OR = 2.023; 95% CI: 1.053–3.884; *p* < 0.05); therefore, maintaining adequate normothermia during the procedure may promote better clinical outcomes and reduce healing complications.	Observational study. Small sample size.
Ziolkowski N, et al. [29]	Canada	Retrospective cohort	To evaluate the association between hypothermia and operative time with postoperative complications in acute burn surgery	In patients with extensive burns, hypothermia predisposed them to infectious complications (RR 1.3; 1.1–1.5; *p* < 0.0017) and non-infectious complications (RR 1.7; 1.2–2.5; *p* < 0.0049). Risk stratification revealed that hypothermic patients with extensive burns undergoing prolonged surgery had a higher risk of infectious complications (RR 1.4; 1.1–1.7; *p* < 0.0068) and non-infectious complications (RR 1.8; 1.1–3.0; *p* < 0.0132) compared with those without these risk factors.	Observational study Small sample size
Fahim M, et al. [30]	United States	Retrospective cohort	To assess whether maintaining normothermia as part of perioperative temperature management strategies is effective in reducing surgical site infections and postoperative complications in patients undergoing colorectal cancer surgery.	Multivariate analysis did not show an association between intraoperative hypothermia and complications, mortality, or readmission, with a surgical site infection rate of 10% at 30 days.	Observational study Small sample size
Eng OS, et al. [31]	United States	Retrospective cohort	To investigate the association between perioperative hypothermia and surgical site infections in patients undergoing cytoreductive surgery with hyperthermic intraperitoneal chemotherapy.	Hypothermia is associated with surgical site infections; in the multivariate analysis, the percentage of surgical time spent in hypothermia was the only associated factor (OR 1.04; 95% CI 1.01–1.07; *p* = 0.008) with surgical site infections within 30 days after surgery.	Single-center study Small sample size
Flores-Maldonado A, et al. [32]	Mexico	Prospective cohort	To assess whether mild perioperative hypothermia is associated with surgical site infection in patients undergoing cholecystectomy.	Hypothermia was found to be a significant independent risk factor for infection (RR 6.3; *p* = 0.01).	Single-center study Small sample size
Seamon MJ, et al. [33]	United States	Retrospective cohort	To determine whether intraoperative hypothermia predisposes patients to postoperative surgical site infections following traumatic laparotomy.	Multivariate analysis determined that a single intraoperative temperature measurement below 35 °C independently increased the risk of site infection by 221% for each degree below 35 °C (OR 2.21; 95% CI: 1.24–3.92, *p* = 0.007).	Single-center study Small sample size
Liedl HJC, et al. [34]	United States	Retrospective cohort	To determine the association between perioperative hypothermia and surgical site infection in patients with diabetes mellitus undergoing elective orthopaedic surgery and non-urgent fracture management.	Perioperative hypothermia is not an independent risk factor for surgical site infection; however, in patients with elevated HbA1c, it was associated with an increased risk of SSI (OR 2.39; 95% CI 1.12–5.32; *p* = 0.022), suggesting an additive effect in the context of poor glycemic control.	Single-center study Small sample size
Kim SH, et al. [35]	Republic of Kore	Retrospective cohort	To determine the effect of intraoperative warming devices on surgical site infection rates in patients undergoing posterior lumbar spinal fusion.	The incidence of surgical site infection was higher in patients who underwent forced-air warming than in those who did not undergo active warming (odds ratio [OR], 1.73; *p* = 0.039), particularly in those over 70 years of age (OR, 4.11; *p* = 0.014).	They compare warming devices but not the relationship between hypothermia and surgical site infection.
Walz JM, et al. [36]	United States	Retrospective cohort	To assess the impact of preoperative antibiotic administration, intraoperative transfusion of blood products, and intraoperative hypothermia on the incidence of surgical site infection in patients undergoing intestinal surgery.	Patients with a lower intraoperative temperature nadir had a lower risk of surgical site infection (*p* = 0.05; odds ratio, 1.33), although this difference is not statistically significant (35.8 ± 0.8 °C vs. 36.0 ± 0.9 °C, *p* < 0.05).	It does not evaluate hypothermia as an independent risk factor for surgical site infection.
Anannamcharoen S, et al. [37]	Thailand	Prospective cohort	To identify factors that increase the risk of incisional surgical site infection for colorectal surgery.	Postoperative hypothermia was identified as an independent risk factor associated with a higher probability of incisional surgical site infection (OR = 5.6; 95% CI: 1.112–28.482; *p* = 0.037).	Single-center study Small sample size
Kurz A, et al. [38]	United States	Randomized clinical trial	To test the hypothesis that hypothermia increases susceptibility to surgical site infection and prolongs hospital stay in patients undergoing colorectal surgery	The final intraoperative core temperature was 34.7 ± 0.6 °C in the hypothermia group and 36.6 ± 0.5 °C in the normothermia group (*p* < 0.001). Surgical site infection occurred in 18 of 96 patients (19%) with hypothermia compared with 6 of 104 (6%) with normothermia (*p* = 0.009). Furthermore, in the hypothermia group, suture removal was delayed by 1 day (*p* = 0.002) and the hospital stay was prolonged by 2.6 days (20%) (*p* = 0.01).	Does not evaluate multiple perioperative confounding factors simultaneously. Follow-up focused on immediate postoperative outcomes.
Shao L, et al. [39]	Turkey	Prospective cohort	To analyze body temperature, immune function, and wound infection rates in patients undergoing open surgery for gastric cancer.	No intergroup differences were found in infection rates one week after surgery.	Limited sample size Single-center study
Kawaraguchi, Y, et al. [40]	Japan	Retrospective cohort	To assess the effect of mild hypothermia on the incidence of surgical site infection and the length of hospital stay in patients undergoing intracranial operation	Surgical site infection was found in 4 of 122 patients (3.3%) in the Hypothermia group, but in none of the 51 patients (0%) in the Normothermia group; however, there were no statistically significant differences in the incidence of surgical site infection or in the length of hospital stay.	Limited sample size
Beilin, B, et al. [41]	Israel	Randomized clinical trial	To determine whether mild perioperative hypothermia affects the cellular immune response in patients undergoing abdominal surgery	The results showed that mild perioperative hypothermia suppresses cellular immune function.	It discusses immune suppression but does not directly address the risk of surgical site infection
Jildeh TR, et al. [42]	United States	Retrospective cohort	To determine the incidence of intraoperative hypothermia in patients undergoing shoulder arthroplasty and its effect on perioperative complications.	Hypothermia showed no significant association with surgical site infections or any other perioperative complications. The incidence of intraoperative hypothermia was 52.7%, advanced age (*p* = 0.002), lower body mass index (*p* = 0.006), the use of interscalene anesthesia (*p* = 0.004), and a lower white blood cell count (*p* < 0.001) were associated with a higher incidence of hypothermia.	Single-center study.
Mohib Y, et al. [43]	Pakistan	Retrospective cohort	To evaluate the incidence of hypothermia in total hip and knee arthroplasty and its relationship to periprosthetic joint infection.	In patients undergoing joint replacement, the incidence of hypothermia was 11.57% and that of infection was 4.2%; only one patient with hypothermia in the total knee replacement group developed an infection (*p* = 0.37), while none in the total hip replacement group did so, and the association with diabetes (*p* = 0.32). Hypothermia was not a risk factor for wound infection following joint replacement.	Limited sample size
Todd M et al. [44]	United States (multicenter)	Randomized clinical trial	To evaluate whether intraoperative hypothermia (33 °C) improves neurological outcomes compared with normothermia in patients undergoing surgery for intracranial aneurysm.	No significant differences were observed between groups in overall adverse events or major outcomes. Surgical site infections were not a primary endpoint; however, infection-related outcomes (e.g., bacteremia) were reported, with a slightly higher incidence in the hypothermia group (5% vs. 3%, *p* = 0.05).	Surgical site infection was not a predefined primary or secondary outcome. The study population was limited to patients with subarachnoid hemorrhage and good neurological grade, which may limit generalizability.

Notes: SSI: surgical site infection; OR: odds ratio; aOR: adjusted odds ratio; RR: relative risk; 95% CI: 95% confidence interval; AUC: area under the curve; HbA1c: glycated hemoglobin; *p*: statistical significance level.

**Table 2:** Operational definitions and measurement approaches of perioperative hypothermia across included studies.

Study	Temperature Threshold	Measurement Type	Exposure Metric	SSI Outcome
Yilmaz Eker, et al. [17]	<36 °C	Not specified	Dichotomous (yes/no)	Primary
Walters, MJ, et al. [18]	≤35.4 °C	Mean intraoperative	Continuous (per 0.5 °C decrease)	Primary
Zeba, S, et al. [19]	Not specified	Not specified	Group comparison (warming vs. control)	Secondary (immune markers)
Zhou YD, et al. [20]	<36 °C	Not specified	Categorical exposure levels	Primary
Pang QY, et al. [21]	<36 °C/>37.3 °C	Repeated measures	Duration + AUC	Primary
Abugri BO, et al. [22]	<36 °C	Not specified	Dichotomous	Primary
Baucom RB, et al. [23]	<36 °C	Not specified	Dichotomous (any hypothermia)	Primary
Siddiqiui T, et al. [24]	<36 °C	Not specified	Dichotomous	Primary
Ribeiro JC, et al. [25]	<36 °C	Repeated measures	Duration (>75 min)	Primary
Tsuchida T, et al. [26]	Not specified	Not specified	Severity categories	Primary
Frisch NB, et al. [27]	Not specified	Not specified	Dichotomous	Primary
Andersen ES, et al. [28]	<35.5 °C	Not specified	Dichotomous + duration	Primary
Ziolkowski N, et al. [29]	Not specified	Not specified	Dichotomous + operative time	Primary
Fahim M, et al. [30]	Not specified	Not specified	Dichotomous	Primary
Eng OS, et al. [31]	<36 °C	Repeated measures	% operative time	Primary
Flores-Maldonado A, et al. [32]	<36 °C	Not specified	Dichotomous	Primary
Seamon MJ, et al. [33]	<35 °C	Single measurement	Continuous (per degree)	Primary
Liedl HJC, et al. [34]	Not specified	Not specified	Dichotomous	Primary
Kim SH, et al. [35]	Not applicable	Not specified	Warming strategy (indirect)	Secondary
Walz JM, et al. [36]	Not specified	Nadir temperature	Continuous	Secondary
Anannamcharoen S, et al. [37]	Not specified	Postoperative	Dichotomous	Primary
Kurz A, et al. [38]	~34.7 °C vs. 36.6 °C	Continuous monitoring	Group comparison	Primary
Shao L, et al. [39]	Not specified	Not specified	Group comparison	Primary
Kawaraguchi, Y, et al. [40]	Not specified	Not specified	Dichotomous	Primary
Beilin, B, et al. [41]	Not specified	Not specified	Immune response	Secondary
Jildeh TR, et al. [42]	<36 °C	Not specified	Dichotomous	Primary
Mohib Y, et al. [43]	<36 °C	Not specified	Dichotomous	Primary
Todd M et al. [44]	33 °C	Controlled	Group comparison	Secondary

Notes: AUC, area under the curve; SSI, surgical site infection; min, minutes; °C, degrees Celsius; %, percentage.

## Data Availability

No new data were created or analyzed in this study.

## Supplementary Materials

The following supporting information can be downloaded at: https://www.mdpi.com/article/10.3390/jcm15124501/s1, Supplementary File S1. Search Strategies (search updated on 30 April 2026); Supplementary File S2. PRISMA Extension for Scoping reviews (PRISMA-ScR) 2018 Checklist.
