# Supplementary material for: Current Evidence on the Relationship Between Perioperative Hypothermia and Surgical Site Infection: A Scoping Review
Source: J Clin Med. 2026 Jun 10;15(12):4501. doi: 10.3390/jcm15124501 (PMC13300808; doi:10.3390/jcm15124501)
Supplement: Supplementary file 1 [file jcm-15-04501-s001.zip › jcm-4257468-supplementary.pdf]

Supplementary File S1. Search Strategies (search updated on 30 April 2026)

PubMed/MEDLINE - 273 results
("Hypothermia"[Mesh] OR hypothermia OR "inadvertent hypothermia" OR "perioperative hypothermia" OR "intraoperative hypothermia" OR "postoperative hypothermia" OR "body temperature" OR "temperature management") AND ("Surgical Wound Infection"[Mesh] OR "surgical site infection" OR "surgical wound infection" OR "surgical infection" OR SSI OR "wound infection" OR "postoperative infection*") AND ("Surgical Procedures, Operative"[Mesh] OR surgery OR surgical OR perioperative OR intraoperative OR operative OR "surgical procedure*") AND ("Randomized Controlled Trial"[Publication Type] OR "Controlled Clinical Trial"[Publication Type] OR randomized OR randomised OR randomly OR trial OR groups) NOT (animals[Mesh] NOT humans[Mesh])<br>Filters: from 1000/1/1 - 2026/4/30
Scopus - 763 results
(TITLE-ABS-KEY (hypothermia OR "inadvertent hypothermia" OR "perioperative hypothermia" OR "intraoperative hypothermia" OR "postoperative hypothermia" OR "body temperature" OR "temperature management")) AND (TITLE-ABS-KEY ("surgical site infection" OR "surgical wound infection" OR "surgical infection" OR "wound infection" OR SSI OR "postoperative infection*")) AND (TITLE-ABS-KEY (surgery OR surgical OR perioperative OR intraoperative OR operative OR "surgical procedure*")) AND (TITLE-ABS-KEY (random* OR trial OR placebo OR "controlled study"))<br>PUBYEAR > 1963 AND PUBYEAR < 2026
Embase – 910 results
(('hypothermia'/exp OR hypothermia OR 'inadvertent hypothermia' OR 'perioperative hypothermia' OR 'intraoperative hypothermia' OR 'postoperative hypothermia' OR 'body temperature'/exp OR 'temperature management')<br>AND ('surgical wound infection'/exp OR 'surgical site infection' OR 'surgical wound infection' OR 'surgical infection' OR ssi OR 'wound infection' OR 'postoperative infection*')<br>AND ('surgical procedure'/exp OR surgery OR surgical OR perioperative OR intraoperative OR operative OR 'surgical procedure*')<br>AND ('randomized controlled trial'/exp OR 'controlled clinical trial'/exp OR random* OR trial OR placebo OR 'controlled study')<br>AND [1964-2025]/py

Supplementary file S2. PRISMA Extension for Scoping reviews (PRISMA-ScR) 2018 Checklist[1]

Section/topic	#	PRISMA-ScR Checklist item	Reported on page #
**TITLE**			
Title	1	Identify the report as a scoping review.	1
**ABSTRACT**			
Structured summary	2	Provide a structured summary including, as applicable: background; objectives; data sources; study eligibility criteria, participants, and interventions; study synthesis methods; results; limitations; conclusions and implications of key findings.	1-2
**INTRODUCTION**			
Rationale	3	Describe the rationale for the review in the context of what is already known. Explain why the review question(s)/objective(s) lend themselves to a scoping review approach.	2-3
Objectives	4	Provide an explicit statement of the question(s) and objective(s) being addressed with reference to their key elements (e.g., population or participants, concepts and context), or other relevant key elements used to conceptualize the review question(s) and/or objective(s)).	2-3
**METHODS**			
Protocol and registration	5	Indicate if a review protocol exists, if and where it can be accessed (e.g., Web address), and, if available, provide registration information including registration number.	3
Eligibility criteria	6	Specify the characteristics of the sources of evidence (e.g., years considered, language, publication status) used as criteria for eligibility, and provide a rationale.	3-4
Information sources	7	Describe all information sources (e.g., databases with dates of coverage, contact with study authors to identify additional sources) in the search and date last searched.	3-4
Search	8	Present full electronic search strategy for at least one database, including any limits used, such that it could be repeated.	Supplementary file
Selection of sources of evidence	9	State the process for selecting studies (i.e., screening, eligibility) included in the scoping review.	3-4
Data charting process	10	Describe the methods of charting data from the included sources of evidence (e.g. piloted forms; forms that have been tested by the team before their use, whether data charting was done independently, in duplicate) and any processes for obtaining and confirming data from investigators.	3-4
Data items	11	List and define all variables for which data were sought and any assumptions and simplifications made.	4-5
Critical appraisal of individual	12	**If done,** provide a rationale for conducting a critical appraisal of included sources of evidence; describe the methods used and how	NA

Section/topic	#	PRISMA-ScR Checklist item	Reported on page #
sources of evidence		this information was used in any data synthesis (if appropriate).	
Summary measures	13	Not applicable for scoping reviews.	NA
Synthesis of results	14	Describe the methods of handling and summarizing the data that were charted.	4-5
Risk of bias across studies	15	Not applicable for scoping reviews.	NA
Additional analyses	16	Not applicable for scoping reviews.	NA
**RESULTS**			
Selection of sources of evidence	17	Give numbers of studies screened, assessed for eligibility, and included in the review, with reasons for exclusions at each stage, ideally using a flow diagram.	Figure 1
Characteristics of sources of evidence	18	For each source of evidence, present characteristics for which data were charted and provide the citations.	Table 1 y 2
Critical appraisal within sources of evidence	19	If done, present data on critical appraisal of included sources of evidence (see item 12).	NA
Results of individual sources of evidence	20	For each included source of evidence, present the relevant data that were charted that relate to the review question(s) and objective(s).	Table 1 y 2
Synthesis of results	21	Summarize and/or present the charting results as they relate to the review question(s) and objective(s).	5-7
Risk of bias across studies	22	Not applicable for scoping reviews.	NA
Additional analysis	23	Not applicable for scoping reviews.	NA
**DISCUSSION**			
Summary of evidence	24	Summarize the main results (including an overview of concepts, themes, and types of evidence available), explain how they relate to the review question(s) and objectives, and consider the relevance to key groups	7-9
Limitations	25	Discuss the limitations of the scoping review process.	10
Conclusions	26	Provide a general interpretation of the results with respect to the review question(s) and objective(s), as well as potential implications and/or next steps.	11
**FUNDING**			
Funding	27	Describe sources of funding for the included sources of evidence, as well as sources of funding for the scoping review. Describe the role	NA

Section/topic	#	PRISMA-ScR Checklist item	Reported on page #
		of the funders of the scoping review.	

Tricco AC, Lillie E, Zarin W, et al. PRISMA Extension for Scoping Reviews (PRISMA-ScR): Checklist and Explanation. *Ann Intern Med* 2018;169(7):467-73.
